# Management of Hypertrophic non-Union After Isolated Fibular Shaft Fracture With Percutaneous Screw Fixation: A Case Report

**DOI:** 10.7759/cureus.74017

**Published:** 2024-11-19

**Authors:** Christos Kotsias, Christos Konstantinidis, Vasileios Panagiotopoulos, Dimitrios Vardakas, Dimitrios Giotis

**Affiliations:** 1 Orthopaedic Department, General Hospital of Ioannina "G. Hatzikosta", Ioannina, GRC

**Keywords:** diaphysis, fibula, fixation, nonunion, pseudoarthrosis, screw

## Abstract

Hypertrophic non-union, after an isolated fibular fracture with intact tibia, is an extremely uncommon complication. The aim of the current study is to present an infrequent case of hypertrophic non-union after an isolated fracture in the proximal diaphysis of fibula which was treated surgically. A 23-year-old male patient presented to our hospital with persistent pain on the lateral aspect of his right leg. He had sustained an oblique isolated fracture in the proximal diaphysis of the fibula after a direct blow to the tibia nine months previously, during a football game, which had been treated conservatively. A hypertrophic non-union was identified on radiographs. Initially, a weight-bearing (WB) cast was placed for one month. However, no sign of union was visible in the new X-rays whereas the symptoms had not subsided. Therefore, a percutaneous osteosynthesis was performed under fluoroscopy with a 3.5 mm lag screw. Immediate mobilization and partial weight-bearing was recommended postoperatively. At six weeks, full WB was allowed, while at eight weeks, radiographic imaging displayed complete union. At 12 weeks postoperatively, the patient returned to his daily routine without any symptoms. Only few studies in literature report non-union as a complication after isolated fibular fractures. In such cases, minimally invasive percutaneous osteosynthesis with a lag screw can offer a successful and definite outcome.

## Introduction

Isolated fibular fractures with intact tibia are very rare and only sporadic cases have been reported in the literature [1]. As the fibula is not a weight-bearing bone, most of these fractures will heal predictably and well without an operation or will be misdiagnosed due to a lack of serious symptomatology [2,3]. Moreover, non-union after a single fibula fracture is an extremely uncommon complication [4]. In the majority of cases, it is completely asymptomatic and the diagnosis might be the result of an incidental radiological examination [2]. However, it could become symptomatic in individuals who participate in physically high-loading activities such as athletes and that is why the true incidence of fibular non-union is difficult to determine. Yet, it is documented at around 0.3% in the literature [1,2].

The most common site of fracture non-union involve fractures of the distal and middle third of the fibula [1,2]. Insufficient blood supply or mechanical instability have been considered as factors that could impair the biological or mechanical environment at the fracture site, respectively, leading to aseptic pseudoarthrosis [2]. In case of symptomatic fibular non-unions, several surgical procedures have been proposed for treatment, including external fixation systems, internal fixation with compression plating and bone graft as well as partial fibulectomy [5].

The aim of the current study is to present an infrequent case of hypertrophic non-union after an isolated fracture in the proximal diaphysis of the fibula which was treated successfully with percutaneous screw fixation. The postoperative management of the case is also highlighted.

This article was previously presented as a meeting abstract at the XXXVI World Congress of Sports Medicine on September 23-26, 2021.

## Case presentation

A 23-year-old man was admitted to our hospital with persistent pain on the lateral aspect of his right leg and had difficulty walking. Nine months ago, he had sustained an oblique isolated fracture in the proximal diaphysis of the fibula after a direct blow to the lateral aspect of the leg during a football game, which had been treated without an operation. No other past medical history was reported. Physical examination revealed local tenderness over the fibular fracture site without muscle atrophy or skin texture. The affected lower limb was neurovascularly intact without any sensory deficits, especially in terms of the common peroneal nerve, and full range of motion for both knee and ankle could be achieved.

**Figure 1:**
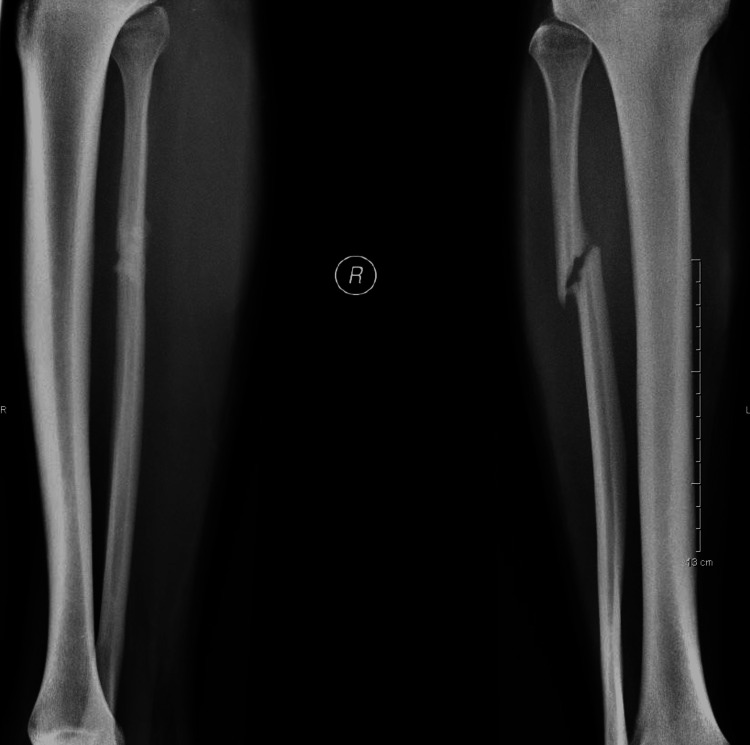
Initial X-rays where the hypertrophic non-union of the fibula can be observed

Radiologic evaluation demonstrated hypertrophic non-union of the proximal diaphysis of the fibula (Figure 1).

**Figure 2:**
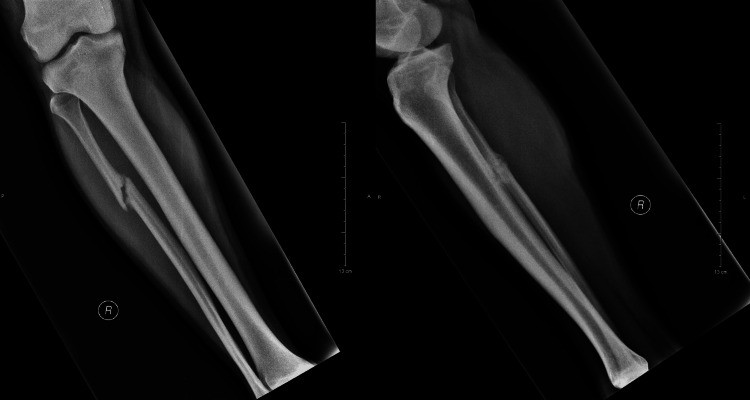
X-rays taken one month after the use of a weight-bearing cast

Initially, nonoperative management was preferred and a weight-bearing cast was placed for one month. However, at the follow-up visit, his symptoms had not subsided and the new radiographs showed no signs of union (Figure 2).

**Figure 3:**
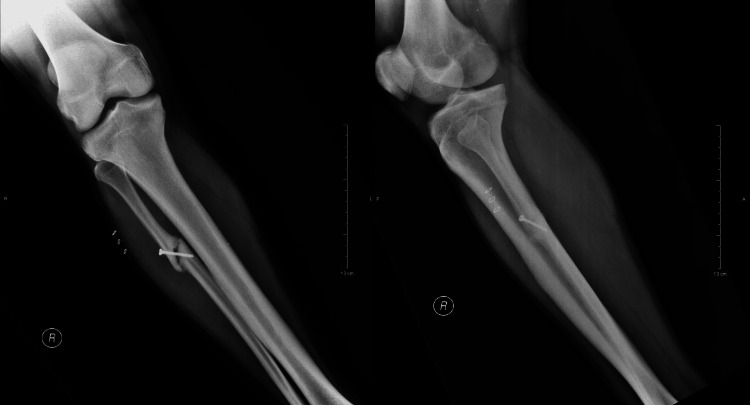
Postoperative radiographs depicting fixation with a lag screw

Therefore, a decision to operate was made which included a minimally invasive approach and percutaneous osteosynthesis of the oblique hypertrophic lesion. Under general anesthesia, a small incision of 2 cm was made and a percutaneous 3.5 mm lag screw was used for fracture site compression under the guidance of real-time fluoroscopy (Figure 3). A protective sleeve was employed to safeguard the neurovascular structures, while drill bits sized 3.5 mm and 2.5 mm were used for the near and far cortex, respectively. Additionally, the screw that was used was a self-tapping screw.

**Figure 4:**
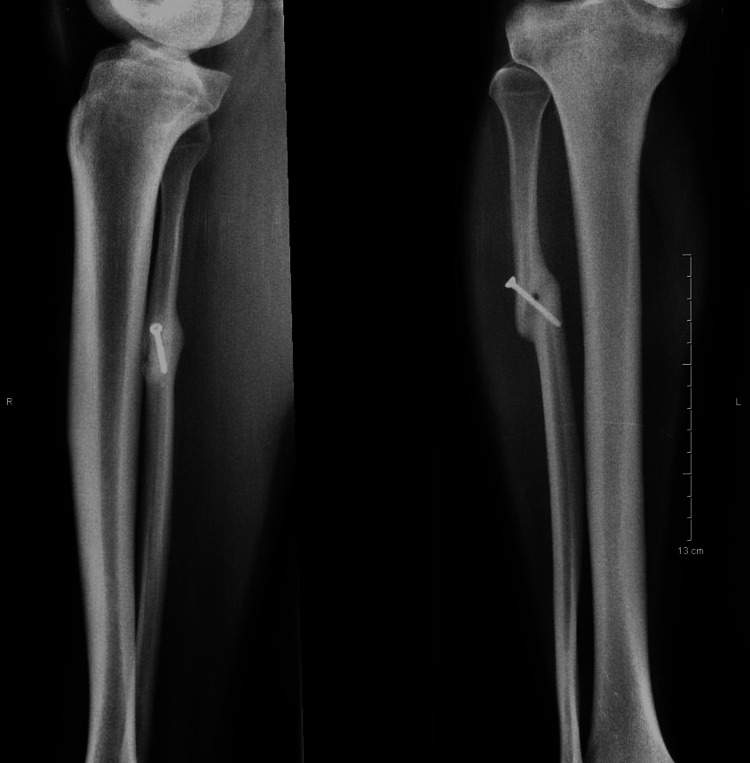
X-rays at eight weeks postoperatively depicting the complete union

The patient was discharged the day after the operation with the administration of a prophylactic dose of low molecular weight heparin for 10 days. He was mobilized immediately after the operation with crutches while partial weight-bearing was recommended as tolerated. Active exercises were also initiated during the patient`s stay in the hospital, followed by an accelerated rehabilitation protocol in order to regain full functional strength. At six weeks, full weight-bearing was allowed whereas at eight weeks, the radiographs displayed a complete union (Figure 4). At 12 weeks, the patient returned to his pre-injury recreational activities and was completely asymptomatic.

## Discussion

Isolated fibular shaft fractures, without syndesmotic ankle injury or concurrent tibial fracture, are rarely reported in the literature [6]. In fact, there are only a few reports of fibular stress fractures in long distance runners as well as insufficiency fractures due to osteoporosis [7,8]. In our case, the patient had neither previous medical conditions nor pre-existing pain in the fibula which could indicate a stress fracture.

The most common mechanism of fibular shaft fractures is a direct blow to the lateral aspect of the tibia [9]. They often heal without any complications since the fibula shaft is almost completely covered by soft tissues and also receives less severe mechanical stresses as compared to the tibia [1,8]. However, non-union, which is believed to be underreported, can occur since it frequently ends up being asymptomatic in the general population [1,2]. In contrast, symptoms manifest mostly in competitive athletes who engage in rigorous physical activities [5,9].

In general, most fibular hypertrophic non-unions do not cause severe symptomatology and therefore normally do not need treatment [9]. However, a small percentage of non-unions might cause severe symptoms, including local pain, swelling, tenderness at the fracture site or even difficulty walking which might require an operation [2]. In the present case, it is more likely that the non-union was the result of mechanical etiology as the existing fibular shaft fracture was not given the opportunity to unite due to continued daily weight-bearing activities of our young patient.

Moreover, according to Bhadra et al., the most common sites of fracture non-unions are the distal and middle third of the fibula probably due to factors related to less soft tissue coverage, less blood supply and increased micromotion [2]. On the contrary, it has been observed that the risk of non-union at the proximal third of the fibula is very low, presumably since this area is better enveloped by muscle and soft tissue [2].

As far as treatment options are concerned, non-operative management has been used in the literature. This has eventually led to delayed fibular union in several cases [2]. Electromagnetic stimulation at the fracture site has been described as a means of accelerating the healing but outcomes still remain controversial [2]. In a symptomatic fibular non-union, when nonoperative treatment fails, surgical management is required.

After reviewing the literature, it was observed that mainly two kinds of treatment are implemented for fibular hypertrophic non-union: open reduction and internal fixation with compression plating and external fixation involving the Illizarov method, both following bone grafting [10,11]. Yet, in our patient, taking into consideration his young age and the need for early recovery as an athlete, we decided against using either of these methods. Since the non-union was oblique, we opted for a minimally invasive technique by achieving compression with a single lag screw. Bone graft was not considered necessary since the non-union was hypertrophic. 

In addition, other techniques including bone graft alone as well as partial fibulectomy have been used for symptomatic fibular non-union [2]. Advocates of fibulectomy state that it is a very cost-effective procedure where the insertion of orthopedic implants is averted, offering a definitive solution without the uncertainty of bony union after operative fixation or bone grafting [5]. However, there are reports of residual pain at the fracture site and clinical instability in the ankle joint after this procedure [12-14]. Therefore it should be used with great caution in patients with high demand such as athletes and only as a last resort after the failure of implants or in cases of septic non-unions [5].

## Conclusions

Hypertrophic non-union of the fibular shaft is an uncommon complication following isolated diaphyseal fibula fractures and is rarely documented in the literature. Minimally invasive percutaneous osteosynthesis using a lag screw is a safe and effective treatment option for hypertrophic non-unions in oblique fractures, though not suitable for transverse or comminuted fractures. Conversely, symptomatic atrophic non-unions of the fibula would require bone grafting with a neutralization plate for oblique fractures or bone grafting with compression plating in transverse fractures.
